# Letter to the editor: is HIF-1α a viable prognostic indicator in OSCC? A critical review of a meta-analysis study

**DOI:** 10.1186/s12957-018-1408-4

**Published:** 2018-06-18

**Authors:** Rama Jayaraj, Chellan Kumarasamy, Madhav Madurantakam Royam, Arikketh Devi, Siddharta Baxi

**Affiliations:** 1 College of Health and Human Sciences, Charles Darwin University, Ellengowan Drive, Darwin, Northern Territory 0909 Australia; 2 School of Bio-Sciences and Technology, Vellore Institute of Technology (VIT), Vellore, Tamil Nadu 632014 India; 3 Department of Genetic Engineering, Kattankulathur Campus, SRM Institute of Science and Technology, Chennai, Tamil Nadu 603203 India; 4 Genesis Cancer Care, Bunbury, Western Australia 6230 Australia

**Keywords:** Letter to the editor, HIF-1α, Oral squamous cell carcinoma, Prognosis

## Abstract

The study performed by Zhou et al. (World J Surg Oncol 15:104, 2017) titled “Clinical and prognostic significance of HIF-1α overexpression in oral squamous cell carcinoma: a meta-analysis” attempts to highlight hypoxia-inducible factor-1 alpha as a possible prognostic marker in oral squamous cell carcinoma (OSCC). We would like to underline a few points which may affect such a conclusion. The correlations between HIF-1α expression and tumour size as well as tumour stage are debatable. Further, the subgroup analysis incorporating Australia and Europe into a single subgroup limits the viability of the prognostic analysis of HIF-1α. We also suggest future studies in the same research area to analyse head and neck squamous cell carcinoma instead of OSCC, to ameliorate the limitations encountered by Zhou et al., due to the scarcity of relevant clinical data and a low number of studies about OSCC.

Dear Editor,

It is well documented and understood that cancer disease progression is intricately linked to the tumour microenvironment [1]. This tumour microenvironment has long been established as a hypoxic environment [2]. Hypoxia-inducible factor-1 alpha (HIF-1α) expression by cells is a standard physiological response to hypoxic environments and is often observed as a systemic response in high-altitude conditions [3]. Considering the hypoxic nature of the tumour microenvironment, overexpression of HIF-1α is an established fact. As the hypoxic environment is found within the tumour mass, expression of HIF-1α increases in proportion to the size and density of a tumour as well as the tumour stage. Zhou et al.’s study [4] establishes an association between tumour size, cancer stage and HIF-1α expression, but with hypoxia as one of the hallmarks of cancer and HIF-1α expression as a physiological response to hypoxia, we would like to indicate that this attempt at association made by Zhou et al. is perhaps redundant.

Secondly, we would like to draw attention to the subgroups chosen for meta-analysis of HIF-1α expression and overall survival (OS). Though the reasoning behind cumulating the studies from Australia and Europe into a single group makes sense, considering the scarcity of studies (a limitation of the study as specified by the authors), such a categorisation, is crude and detracts from the purpose of a subgroup analysis. Subgroup analysis is performed to provide a higher resolution of insight into a meta-analysis and requires classification of the subgroups based on common criteria between studies that may influence the overall outcome effect (in this case, patient survival). If we presume that the classification by Zhou et al. was intended to take into account significant hereditary genetic variations, in the form of race or ethnicity, as affecting overall outcome effect, the continental classification is still an imprecise method of doing so. This is due to a lack of information showing the ethnic distribution of the patient samples. Though we may presume that studies from Europe and Australia primarily involve Caucasian population, other ethnic groups such as the Aboriginal Australians and non-Caucasians may also be part of such studies, thereby making a classification which combines Australia and Europe into a single group, erroneous and inapt.

We would also like to address the limitation of the study, where the authors cited a small pool of eligible studies. As oral squamous cell carcinoma (OSCC) is a highly specific subset of cancer, it severely narrows down the number of studies in the field. A method of ameliorating this problem could be to choose head and neck squamous cell carcinoma (HNSCC) instead of OSCC to perform such a meta-analysis study. As OSCC is a subset of HNSCC, a much larger pool of viable research can be obtained, and due to etiological similarities between OSCC and other types of HNSCC, it is plausible to assume that establishing HIF-1α as a reliable prognostic indicator in HNSCC would imply its prognostic importance/effect in OSCC, as well.

In our opinion, the principal merit of the study is the association achieved between HIF-1α expression and lymph node status and histological differentiation, which provides valuable clinically relevant information. However, we present our remarks not to highlight the limitations of this study but to merely communicate possible improvements in the concept and design to Zhou et al. and other prospective authors in the same discipline, in case of a planned update to this study in a few years, as well as the scientific community at large.

## Abbreviations

HIF-1α: Hypoxia-inducible factor-1 alpha
HNSCC: Head and neck squamous cell carcinoma
OS: Overall survival
OSCC: Oral squamous cell carcinoma
